# Suitability of Slower Growing Commercial Turkey Strains for Organic Husbandry in Terms of Animal Welfare and Performance

**DOI:** 10.3389/fvets.2020.600846

**Published:** 2021-01-06

**Authors:** Anna Olschewsky, Katharina Riehn, Ute Knierim

**Affiliations:** ^1^Farm Animal Behavior and Husbandry Section, Faculty of Organic Agricultural Sciences, University of Kassel, Witzenhausen, Germany; ^2^Department Ecotrophology, Faculty of Life Sciences, Hamburg University of Applied Sciences, Hamburg, Germany

**Keywords:** animal welfare, behavior, health, performance, turkey strains

## Abstract

Intensive turkey production with fast growing strains is often critically discussed regarding animal welfare problems. Studies evaluating the welfare status of both organic and less intensive selected turkey strains are limited, except in the slightly slower growing Kelly Broad Breast Bronze (Kelly). The aim of this study was to assess the welfare of turkeys from two strains with further decreased growth rate, Hockenhull Large Bronze (HoBr) and Hockenhull Black (HoBl), in comparison to Kelly under commercial organic conditions with 100% organic feed. Altogether 844 non-beak-trimmed male turkeys (274–288 per line) were reared and fattened in three replications with each six groups. On group level, use of resources in the 7, 16, and 25th week of life, mortality and feed conversion were recorded. Each bird was assessed with regard to plumage and skin condition as indicators of agonistic interactions, cannibalism and feather pecking, with regard to leg health, footpad, breast skin condition and, as performance indicators, live and carcass weight, utilization, daily weight gain and weights of valuable meat parts. The significantly slower growing HoBl showed slightly fewer malposition of the legs, reduced injury rates and less breast buttons, but a higher susceptibility to footpad dermatitis than Kelly turkeys. HoBr with a similar growth rate compared to Kelly had slightly more problems concerning walking ability and plumage damage, but also less breast buttons than Kelly turkeys. However, effect sizes were negligible (Φ < 0.10), except for the higher occurrence of footpad dermatitis and the reduced number of breast buttons in HoBl with small effect sizes (Φ = 0.20–0.24). Use of resources, prevalence of breast blisters and mortality, were not statistically different, although mortality rate was numerically lower in HoBl. Thus, for none of the studied strains clear benefits or disadvantages in terms of the birds' predisposition for welfare problems could be identified. Overall, prevalences of animal welfare problems were mostly lower than in comparable studies while performances were comparatively high. Therefore, turkeys from the studied strains appear to be suitable for organic rearing and fattening with 100% organic feed, given a good management.

## Introduction

Turkey meat production is increasing worldwide (1). The EU countries produce around 33% of the world's turkey meat (~ 2 million tons in 2018) and are therefore the largest producer following the USA (2). In 2019, Germany was the largest producer in Europe with almost 500,000 tons of turkey meat (2). Most of these turkeys are reared and fattened under conventional conditions, while <2% of the housing places are certified organic (3). In the European Union, organic animal husbandry is regulated by EU-Regulation 889/2008 (4). Among others, the rules shall safeguard higher animal welfare standards, based e.g. on higher space allowances or access to a free-range (4). This regulation further stipulates: “To prevent the use of intensive rearing methods, poultry shall either be reared until they reach a minimum age or else shall come from slow-growing poultry strains”. Since the middle of the 20th century turkey breeding is economically very successful, not at least because of intensive methods of cross-breeding (5). As a result, the live weight of turkeys has quadrupled compared to wild turkeys (6). The use of fast growing turkeys for meat production dominates in Germany as well as in the rest of the world (7).

However, turkey husbandry is often critically discussed because of several potential animal welfare problems. Among them is a high risk for skin injuries and damaged or lost feathers due to agonistic interactions and the behavioral disorders cannibalism and feather pecking (5). Dalton et al. (8) summarized agonistic interactions, cannibalism and feather pecking as injurious pecking. Extensive injuries particularly on top of the head and large feather losses can lead to the death of animals (8). Causes are multifactorial, including genetic, nutritional and husbandry factors (8, 9). Rather similar ranges of affected birds have been reported from investigations of commercial and experimental, conventional and organic conditions, with 77–100% of turkeys with plumage damage (10–12) as well as 23–39% with injuries (10–15). Further animal welfare problems may be respiratory and cardiovascular diseases (5) as well as high prevalences of footpad dermatitis, impaired leg health and breast skin alterations. Again, different European and North American studies of mainly fast, but also slower growing turkeys under commercial, experimental, conventional or organic conditions have found similar ranges of these welfare problems. Footpad dermatitis was present in more than 80% of investigated birds (14, 16–21). Footpad dermatitis can appear in various forms of hyperkeratosis and necrosis due to inflammatory processes (22, 23) and is caused or promoted by a number of factors, with litter moisture playing a decisive role (24, 25). Impaired leg health with reduced walking ability or malposition of the legs can be linked to a multitude of genetic, nutritional and husbandry factors (5, 26). Prevalences of more than 50% of turkeys with reduced walking ability or malposition of the legs were found (10, 27, 28). In addition, breast skin alterations, which are also indicative of impaired welfare (29–31), were present in 8–48% of birds (10, 12, 21, 32–36). These alterations include both breast buttons, which are lesions in unfeathered areas, as well as breast blisters, which are inflammations of the *bursa sternalis*, with breast buttons being the more frequent findings (10, 34, 35).

It is supposed that problems regarding health or behavior in turkeys are mainly attributed to the high growth potential of the commonly used commercial hybrids and the intensive husbandry conditions in conventional farming (5, 36). However, the outcomes in organic husbandry are not fundamentally different. One reason for this may be the widespread use of conventional fast growing turkeys in organic husbandry (3) due to the EU regulation 889/2008 (4) stipulating minimum slaughter ages that are compatible with common strains suitable for cutting, since there is hardly any demand for whole turkeys in Germany (3, 7). Some organic farms, nevertheless, keep a slightly slower growing commercial turkey line, Kelly Broad Breast Bronze (BBB) (3), for which slightly reduced prevalences of welfare problems have been reported (10, 28, 34, 37). The majority of German organic farmers is keeping female turkeys of fast-growing strains, mostly B.U.T Big 6 (3), because of their sex-specific lower nutritional demands, and the lower growth rate which may contribute to a reduced susceptibility to welfare problems (36). This is e.g. reflected by reports of lower prevalences of damaging feather pecking (13) and breast skin alterations (11, 21, 29). However, all the mentioned welfare outcomes are still not satisfactory, considering the high welfare level expected in organic farming (4). Furthermore, it is debatable whether the use of predominantly one sex in organic farming is an acceptable practice. In our opinion the assessment of the suitability of a strain for organic farming should be based on the most challenging condition which is the rearing and fattening of male turkeys with 100% organic feeding.

It was therefore the aim of this study to assess the welfare of male turkeys under organic husbandry conditions in two strains with further decreased growth rate in comparison to Kelly BBB.

## Materials and Methods

### Birds and Husbandry

Three fattening batches (cycles) from July 2015 to January 2018 on a commercial organic farm in Northern Germany were monitored. Rearing and fattening conformed to the Animal welfare - farm animal husbandry ordinance (38), the EU regulation 889/2008 for organic farming (4) as well as to Demeter guidelines (39). Management (climate control, health care, animal controls) was in accordance with standard commercial guidelines (40).

Besides the reference Kelly BBB (Kelly), the strains Hockenhull Large Bronze (HoBr, recommended for free-range husbandry, nearly similar growth potential as Kelly BBB) and Hockenhull Black (HoBl, recommended as robust, lower target live weight, markedly reduced growth potential), both from Aviagen (UK), were used. They had been selected based on enquiries in Germany, UK and France in 2015 considering the criteria (a) lower, but sufficient growth potential, (b) stated robustness, (c) suitability for cutting, and (d) commercial availability. Hockenhull turkeys were delivered from the UK, and Kelly turkeys from the Netherlands by car as non-beak-trimmed, male 1-day-poults. The aim was to rear and fatten 100 turkeys per strain and batch, divided in two groups of 50 individuals. This was repeated twice, so that three batches were conducted. Due to deviations in delivered numbers and sex of birds, altogether 844 male turkeys were investigated instead of the planned 900 birds. The birds were individually marked at the end of rearing, using colored and numbered leg bands. These were changed in the 13th week of life in order to adapt to the birds' growth.

Due to the seasonal availability of HoBl, each batch started at the end of July or beginning of August. Rearing (1–6th week of life) and fattening (7–25th week of life) took place in a mobile house which contained six pens (each measuring 3.5 × 5.0 m) and a central control aisle. The birds had access to separate winter gardens (each 2.5 × 5.0 m) from the 5th week of life onwards. These were attached to the mobile house and had a roof but no solid floor which was, however, littered. On the sides they were covered with wood in the lower part and with windbreak netting in the upper part. The pens inside the house and the winter gardens were separated by wire mesh fences so that the groups could not mix. At the end of the rearing phase the turkeys received access to separate grass-covered free-range areas (500 m^2^ per group) with electric fencing. Each pen was equipped with feeders (small, plastic round feeders in the rearing period and bigger, metal ones in the fattening period, all filled by hand) and round drinkers (1st−3rd week: bell drinkers filled by hand, 4th−25th week: plasson drinkers connected to the regular water supply). Their height was regularly adapted to the growth of the turkeys. In the 2nd week of life, round, wooden perches (Ø 2 cm) were placed in each pen. With the start of the fattening period, they were replaced by larger perches (round metal perches, Ø 4 cm, in the first batch and wooden perches, squared timbers of 3 × 6 cm, in the second and third batch). Additionally, sand was provided inside the pens during the rearing phase, which was replaced by a mixture of grit and sea shells as well as pecking blocks at the beginning of the fattening period. Pens were littered mainly with wood shavings from day 1 until the 25th week of life every couple of days as needed. In the fattening period also straw was used. The house was supplied with regular electricity, ventilation was semi-automatic, and gas radiant heaters provided heating.

Compound feed with 100% organic components was purchased and fed in six feeding phases (Table 1). From the 14th week of life onwards the compound feed was supplemented with increasing proportions of the farm's own wheat (wheat %: 14–15th week = 10%, 16–19th week = 20%, 20–25th week = 30%).

**Table 1:** Average nutrient contents in the rations including supplemented wheat in the different feeding phases over three fattening batches (cycles).

	**Ration 1**	**Ration 2**	**Ration 3**	**Ration 4**	**Ration 5**	**Ration 6**
Week of life	1–3	4–9	10–13	14–15	16–19	20–25
ME-poultry (MJ/kg)	11.4	11.8	11.3	11.4	9.0	7.9
Raw protein (%)	23.9	22.3	19.4	17.5	15.5	13.6
Methionine (%)	0.44	0.43	0.34	0.3	0.3	0.2
Lysine (%)	1.21	1.16	0.89	0.8	0.7	0.6
Sodium (%)	0.21	0.19	0.16	<0.02	<0.02	<0.02
Starch (%)	29.8	31.0	34.5	31.1	27.6	24.2
Fiber (%)	5.0	5.2	6.7	6.0	5.4	4.7

Conforming to the farm's usual practice, the birds were slaughtered consecutively in the 17, 20, and 25th week of life, respectively.

### Recording of Behavioral, Health and Performance Measures

In the 7, 16, and 25th week of life all animals of every group inside the pen that used either the “feeding area” or “perches” were counted via instantaneous scan sampling (41) with a 15-min-interval. Observations were carried out by one person on four consecutive days during each time 4 h (i.e., covering the light period from 9 A.M. to 5 P.M. twice) and noted in check sheets. In parallel, birds using the winter garden were counted in the same way and time intervals (scans) based on videos. Finally, the number of birds using the free-range area was calculated as the rest of birds from the total number minus those recorded in the pen and winter garden. Number of recordings were partly reduced due to free-range closure because of histomonosis infections, a camera failure and removal of perches to prevent animal accidents. Intra- and inter-observer reliability for video recordings and inter-observer-reliability between two persons for direct observations were both good (*r* = 0.89–1.0, *n* = 17 observations of each 2 h).

After the end of the behavioral observations, the physical condition of all birds was assessed. The applied clinical scoring schemes were adapted from earlier studies in order to allow maximal comparability of results (Table 2). In the 7, 16, and 25th week of life, the assessment of walking ability and leg position was conducted: all individuals were carefully encouraged to walk along a path of 3 m for the gait scoring. Then they jumped on a straw bale where their leg position was rated. If a bird refused to move or showed poor locomotion, they were not forced to walk the entire 3 m distance and to jump on the bale. Afterwards they were fixed in upright position by one person on the straw bale or a table (depending on the bird's weight) for the investigation of plumage, skin condition and footpads (by consecutively lifting legs backwards). Their plumage condition at the back, sides and wings and skin injuries at the whole body, except the breast, were scored while stroking back the feathers. For the assessment of the footpads in the 7 and 16th week of life, both footpads were cleaned with a brush and additionally with water, if necessary, before scoring. In case of different findings on both footpads, the worse result was documented. In addition, footpads of all slaughtered animals were examined in the 20 and 25th week of life in the same way. The occurrence of breast blisters and breast buttons was recorded after slaughter and plucking in all birds.

**Table 2:** Scoring schemes, modified after the given references, with definitions, and results of inter-assessor-reliability testing for the measures regarding physical condition.

**Score**	**Plumage (without tail feathers)**	**Skin**	**Gait**	**Leg position**	**Footpads**	**Breast blister**	**Breast button**
0	Completely intact and smooth plumage (including tips of feathers), no bare skin areas^A^	No injuries, up to 3 point-like bruises	Upright, steady striding, toes are bent backwards when the leg is lifted	parallel	Intact skin, no swelling	No breast blister	No breast button
1	Single feathers damaged (pecked, disheveled or broken), no bare skin areas^A^	Superficial, point-like injuries or >3 bruises or bloodied feather follicles	Slight abnormality, foot is quickly put down again after lifting, toes are not bent	x-shaped, smaller distance between hocks than between feet	Hyperceratosis, small necrotic spots or slight swelling concerning footpads or toes	Small round swelling, can be fluctuating	Point-like hardening
2	Several feathers damaged or bare skin areas^A^ ≤ 2.5 cm (largest diameter)	Deeper and larger injuries ≤ 2.5 cm (largest diameter)	Strong pendulum locomotion due to lameness on one or both sides	Wide-legged, the legs are parallel but with a wider distance (leg position at rump at outer side)	Larger necrotic areas <50% of footpad or at ≤ 2 toes or moderately swollen toes	Fist-sized fluctuating or hardened swelling	Skin lesion ≤ 2 cm (largest diameter)
3	Many feathers damaged or bare skin areas^A^ >2.5 −10 cm (largest diameter)	Deep and large injuries >2.5 cm (largest diameter)	Bird sits down again as soon as possible or can only move with great effort (e.g., flapping of wings)	o-shaped, greater distance between hocks than between feet	Large necrotic areas >50% of footpad or at >2 toes or severely swollen toes	Double fist-sized fluctuating or hardened swelling	Skin lesion >2 cm (largest diameter) ≥ 2 cm
4	Large areas with damaged feathers or bare skin^A^ >10 cm (largest diameter)	–	–	–	–	–	–
References	(10–12, 33)	(33)	(10, 12)	(10, 12)	(42)	(10)	(10)
Inter-assessor-reliability (PABAK)	*K* = 0.73–0.90 (*n* = 90)	0.69–1.0 (*n* = 120)	0.90–1.0 (*n* = 60)	0.60–1.0 (*n* = 90)	0.81–0.87 (*n* = 111)	0.87–0.90 (*n* = 30)	0.87–0.90 (*n* = 30)

A Including bare areas with feather follicles visible.

Furthermore, losses were documented, and carcasses subjected to pathological-anatomical examinations by the Veterinary Pathology Department of the University of Leipzig. Also, the results of the official *ante-mortem* and *post-mortem* slaughter-inspections were documented.

Regarding performance, all living turkeys were weighed with a manual poultry scale (BAT1, VEIT electronics with a capacity of max. 50 kg and an accuracy of 1 g) in the 7, 16, and 25th week of life, and additionally, birds intended for slaughter in the 17 and 20th week of life. Individual carcass weights were taken 4 h after evisceration (without head and legs) using a digital scale. Utilization was calculated as percentage of carcass from live weight. In addition, the weights of valuable meat parts (breast, upper and lower legs) were determined from 10% of slaughtered and cutted turkeys. The individual daily weight gains were calculated for the birds slaughtered in the 25th week of life. Feed conversion for each group was calculated based on the provided feed that had been continuously recorded. Back-weighing of feed was conducted each before weighing of the turkeys. Losses of feed inside the pens were not recorded.

For all measures based on scoring, an acceptable to good inter-assessor-reliability between two persons was ascertained (Table 2).

### Statistical Analysis

Data on group level, such as the behavioral data, were analyzed using non-parametric tests due to small sample sizes: Friedmann test was used to ascertain possible time effects over the three observation points of time, independent of treatments (strain), which were compared using Kruskal-Wallis test. Kruskal-Wallis test was also used to analyze possible treatment effects on mortality and feed conversion.

Individual data per animal, in case of ordinal data, were dichotomized by converting score 0 to 0 and score > 0 to 1. Generalized linear mixed models (in r, package lme4 v1.1-21, glmer with glmerControl optimized by Bobyqa) were applied with the fixed factors “strain,” “week of life” and their interaction, and the random effect “animal nested in group and batch” for repeated measurements, or “group nested in batch” for single measurements. Using the package lsmeans v2.30-0 mean and confidence interval averaged over the levels of “week of life” were estimated for binomial data. The reference strain Kelly was defined as intercept in order to allow comparisons with the two other strains. Metric individual data were analyzed in the same way, with the same factors, with linear mixed models (package lme4 v1.1-21, lmer with lmerControl, optimized by Bobyqa and lmerTest v3.1-3). Normal distribution of residuals as well as variance homogeneity were checked in r (QQ-normal plot, skewness, kurtosis and scatter plot) and, in case of live and carcass weight, utilization and lower leg weight, data transformed in r by logarithmizing the square root. Nevertheless, normal distribution could not be reached for the variables live weight, carcass weight and utilization. Therefore, Kruskal-Wallis test and *post-hoc* Mann-Whitney-*U*-test were used. The script for the mentioned calculations in r are listed in Supplementary File 1.

In addition, effect sizes were calculated using SPSS for all test results. According to Ellis (43), phi-correlation (Φ) was computed for dichotomized data and point-biserial correlation (r_pb_) for metric data, respectively.

## Results

Mortality rates did not differ significantly between the strains (*p* = 0.41, χ^2^ = 1.77, df = 2, *n* = 6, r_pb_ = 0.16–0.46). The causes of losses and in some cases necessary cullings of animals are displayed in Table 3.

**Table 3:** Mortality and causes over three fattening batches (cycles).

**Causes of death**	**Kelly BBB**		**Hockenhull bronze**		**Hockenhull black**	
	***n***	**%**	***n***	**%**	***n***	**%**
First 2 weeks of life: unthrifty birds or leg deformities	7	2.4	6	2.1	6	2.1
Accidents	0	0.0	4	1.4	5	1.7
Histomonosis	10	3.4	7	2.5	2	0.7
Cannibalism	1	0.3	2	0.7	0	0.0
Enteritis	1	0.3	2	0.7	1	0.4
Hepatitis	1	0.3	1	0.4	0	0.0
Unknown cause	1	0.3	3	1.1	1	0.4
Total losses	21		25		15	
Total mortality	7.2		8.8		5.2	

Histomonosis was diagnosed by histological and PCR examinations of tissue samples from caecum and liver in all three batches and led to altogether 19 losses (Table 3). However, during the official *post-mortem* slaughter inspections only a few pathologic-anatomical changes were found in the surviving animals.

**Figure 1:**
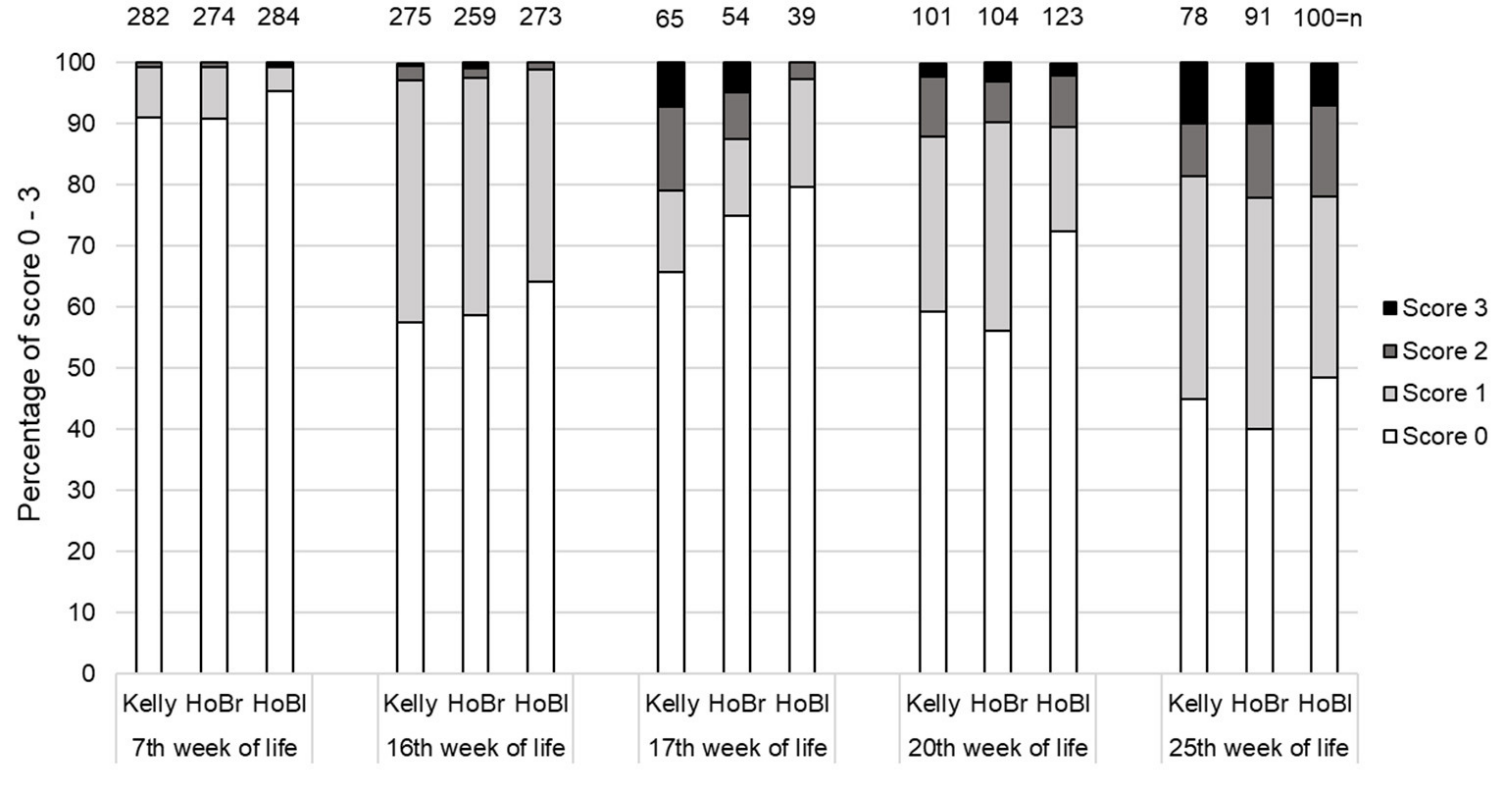
Percentages of turkeys with different skin injury scores (score 0 = intact, score 1 = superficial spots, score 2 < 2.5 cm, score 3 > 2.5 cm) at five scoring times, in three strains (Kelly = Kelly BBB, HoBr = Hockenhull Bronze, HoBl = Hockenhull Black) over three fattening batches (cycles), each with two groups per strain, with n = total number of individuals assessed.

Losses due to cannibalism were recorded once in Kelly and twice in HoBr (Table 3). Kelly had significantly more injuries than HoBl, but not than HoBr (Table 4). The majority of injuries was superficial and point-like (score 1). Score 2 was found in 0.4–15.0% and score 3 in 0.3–10.0% of assessments (Figure 1). Overall, a significant effect of the factor “week of life” was detected (*p* < 0.01) but no significant interaction between “strain” and “week of life.”

**Table 4:** Results of generalized linear mixed models and effect sizes (phi-correlation) regarding possible effects of “strain” and “week of life” on the turkeys' physical condition including estimated mean and confidence interval averaged over the levels of “week of life”; significant interactions are stated in the text (Kelly = Kelly Broad Breast Bronze, HoBr = Hockenhull Bronze, HoBl = Hockenhull Black).

**Measure**	**Line**	**Mean yes (%) CI**	**Est**.	**Std. error**	***z*-value**	***p*-value**	**OR**	**Φ**
Skin injuries (yes vs. no)	Kelly vs.	30.2 16.4; 48.8						
	HoBr	31.0	0.035	0.184	0.191	0.848	1.05	0.01
		16.9; 49.7						
	HoBl	22.2	−0.414	0.187	−2.216	0.027	0.78	−0.06
		11.4; 38.7						
Plumage damage (yes vs. no)	Kelly vs.	88.5 83.9; 91.9						
	HoBr	92.9	1.150	0.541	2.125	0.034	1.49	0.07
		89.0; 95.4						
	HoBl	90.3	−0.053	0.394	−0.135	0.893	1.55	0.08
		86.3; 93.3						
Impaired walking ability (yes vs. no)	Kelly vs.	1.0 0.4; 2.6						
	HoBr	3.0	1.132	0.480	2.360	0.018	2.80	0.10
		13.3; 7.0						
	HoBl	0.9	−0.115	0.517	−0.222	0.825	1.03	0.00
		0.3; 2.3						
Malpositions of legs (yes vs. no)	Kelly vs.	10.5 6.5; 16.3						
	HoBr	8.4	−0.244	0.302	−0.805	0.421	0.85	−0.03
		5.1; 13.6						
	HoBl	5.3 3.1; 9.0	−0.733	0.312	−2.347	0.019	0.55	−0.09
Footpad dermatitis (yes vs. no)	Kelly vs.	24.3 8.0; 54.1						
	HoBr	35.6	0.344	0.448	0.767	0.443	1.56	0.11
		13.1; 66.9						
	HoBl	64.6	1.390	0.439	3.170	0.002	2.68	0.24
		33.4; 87.0						
Breast blister (yes vs. no)	Kelly vs.	2.7 0.8; 8.5						
	HoBr	2.2	−0.244	0.508	−0.480	0.631	0.77	−0.02
		0.6; 7.1						
	HoBl	1.4	−0.663	0.562	−1.181	0.238	0.51	−0.05
		0.4; 5.3						
Breast buttons (yes vs. no)	Kelly vs.	9.8 6.2; 15.1						
	HoBr	3.4	−1.134	0.402	−2.819	0.005	0.33	−0.13
		1.7; 6.8						
	HoBl	1.1	−2.319	0.620	−3.739	<0.001	0.10	−0.20
		0.3; 3.4						

CI, 95% confidence interval; Est., Estimate; Std. error, Standard error; OR, Odds ratio; Φ, Phi-correlation.

**Figure 2:**
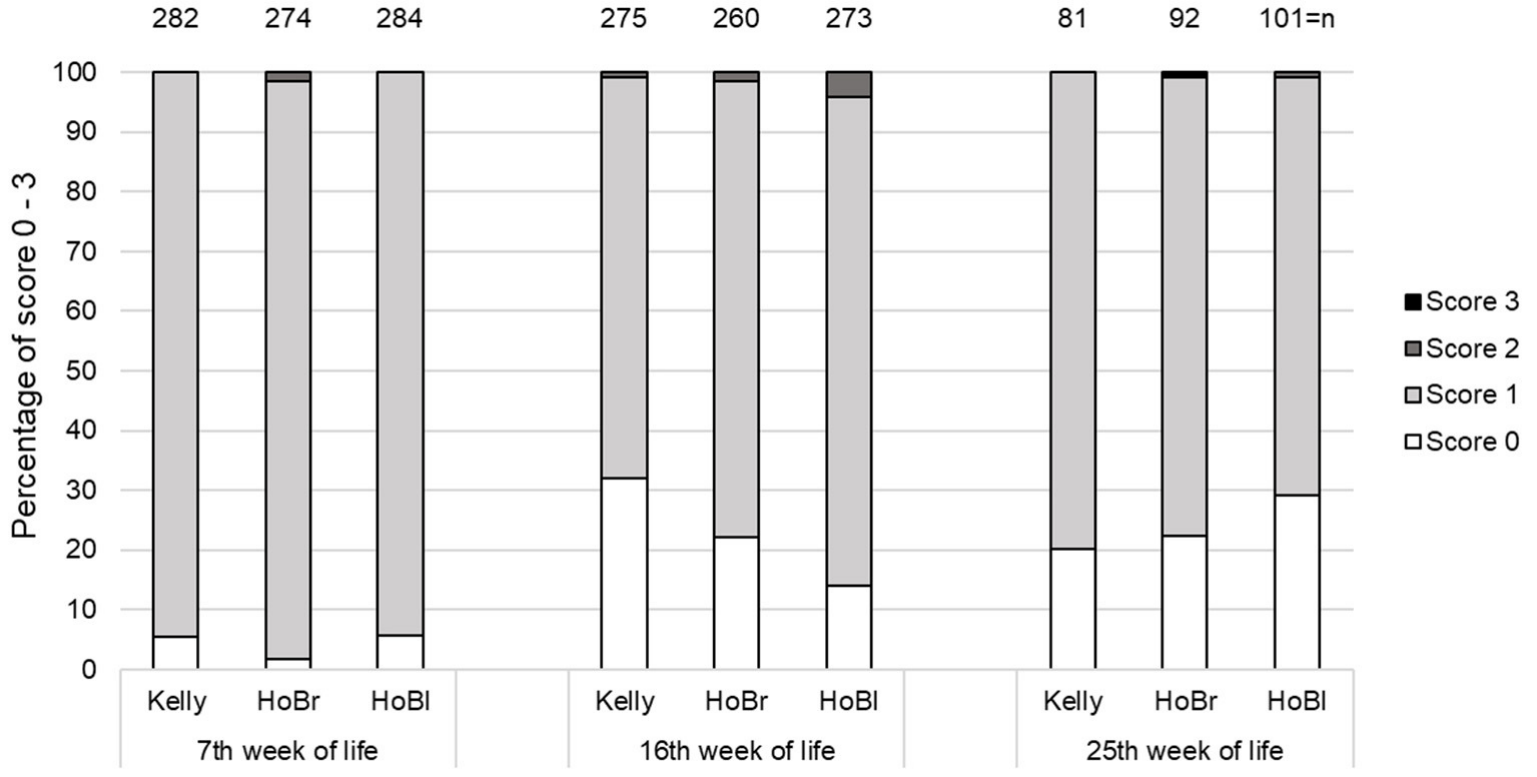
Percentage of turkeys with different plumage condition scores (score 0 = intact, score 1 = single feathers pecked or broken, score 2 = several feathers pecked, broken or smaller bare skin areas, score 3 = larger plumage damage or larger bare skin areas) at three scoring times in three strains (Kelly = Kelly BBB, HoBr = Hockenhull Bronze, HoBl = Hockenhull Black) over three fattening batches (cycles), each with two groups per strain, with n = total number of individuals assessed.

Regarding plumage condition, in all turkeys and at all assessment times predominantly slight damage of single feathers (score 1) was found. Score 4 was never present, score 3 only once and score 2 in 0.8–4.0% of all cases (Figure 2). Significantly less HoBr turkeys had a completely intact plumage than Kelly, while HoBl did not differ (Table 4). However, there were significant interactions between “week of life” and “strain”: The proportion of turkeys with no plumage damage decreased in Kelly between the 16 and 25th week of life, while it increased in HoBl (*p* < 0.01) and changed little in HoBr (*p* = 0.04). Additionally, a significant effect of “week of life” was detected (*p* < 0.01).

**Figure 3:**
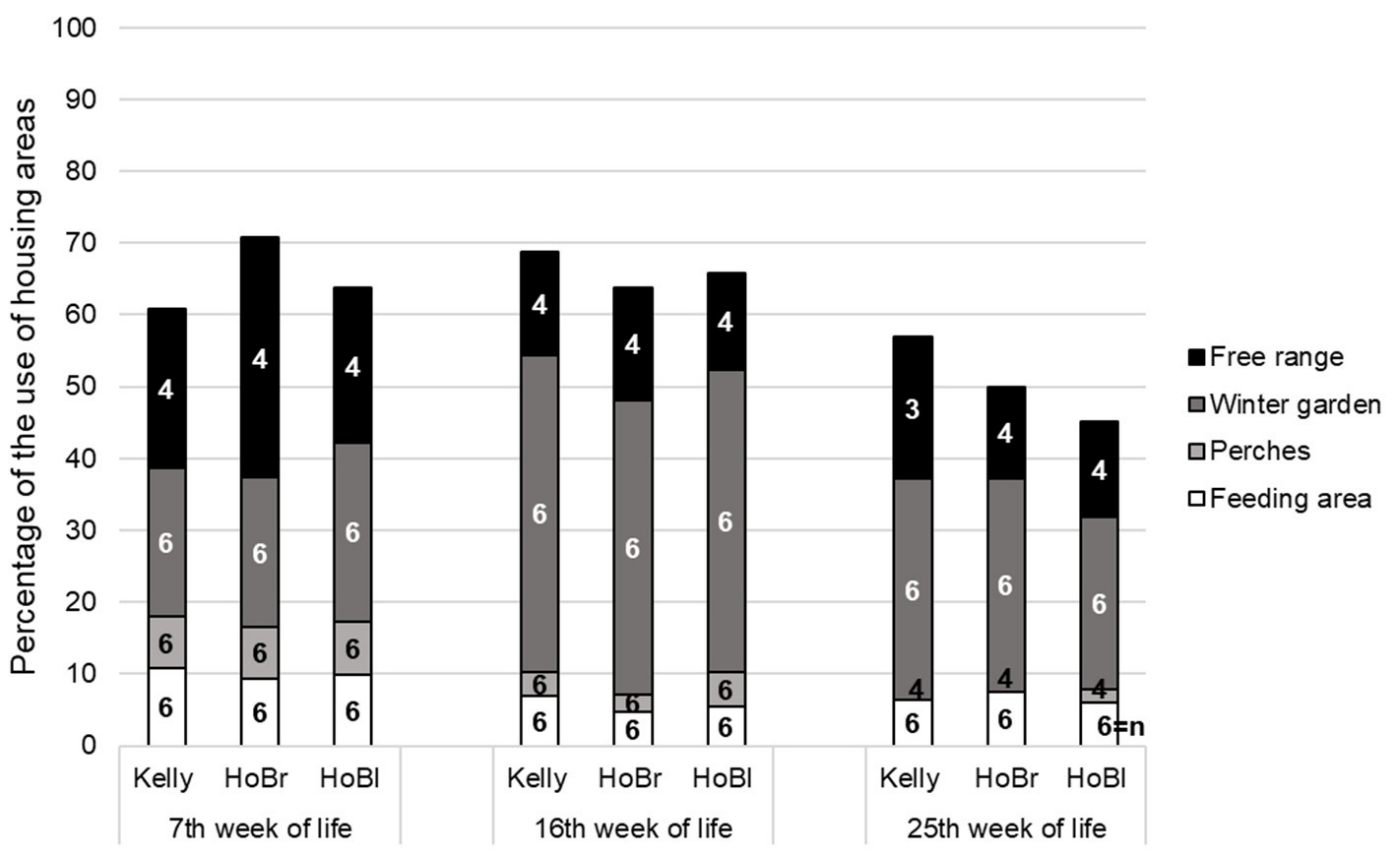
Use of different resources during eight light hours (average percentage of observed time per animal) at three observation time points in three strains (Kelly = Kelly BBB, HoBr = Hockenhull Bronze, HoBl = Hockenhull Black) with *n* = 3 fattening batches (cycles) * 2 groups (partly reduced n: free-range closure due to histomonosis infection, camera failure, removal of perches due to animal accidents).

Time effects on the use of the different resources were identified regarding the feeding area (*p* < 0.01, χ^2^ = 16.78, df = 2, *n* = 18), perches (*p* < 0.01, χ^2^ = 16.89, df = 2, *n* = 12) and winter gardens (*p* < 0.01, χ^2^ = 28.78, df = 2, *n* = 18), but not for the free-range area (*p* = 0.51, χ^2^ = 1.33, df = 2, *n* = 6; Figure 3). No significant differences between the strains were found (analyzed separately for each observation period, except for the free-range area) (*p* = 0.06–0.90, χ^2^ = 0.22–5.55, df = 2, *n* = 4–6; Figure 3).

**Figure 4:**
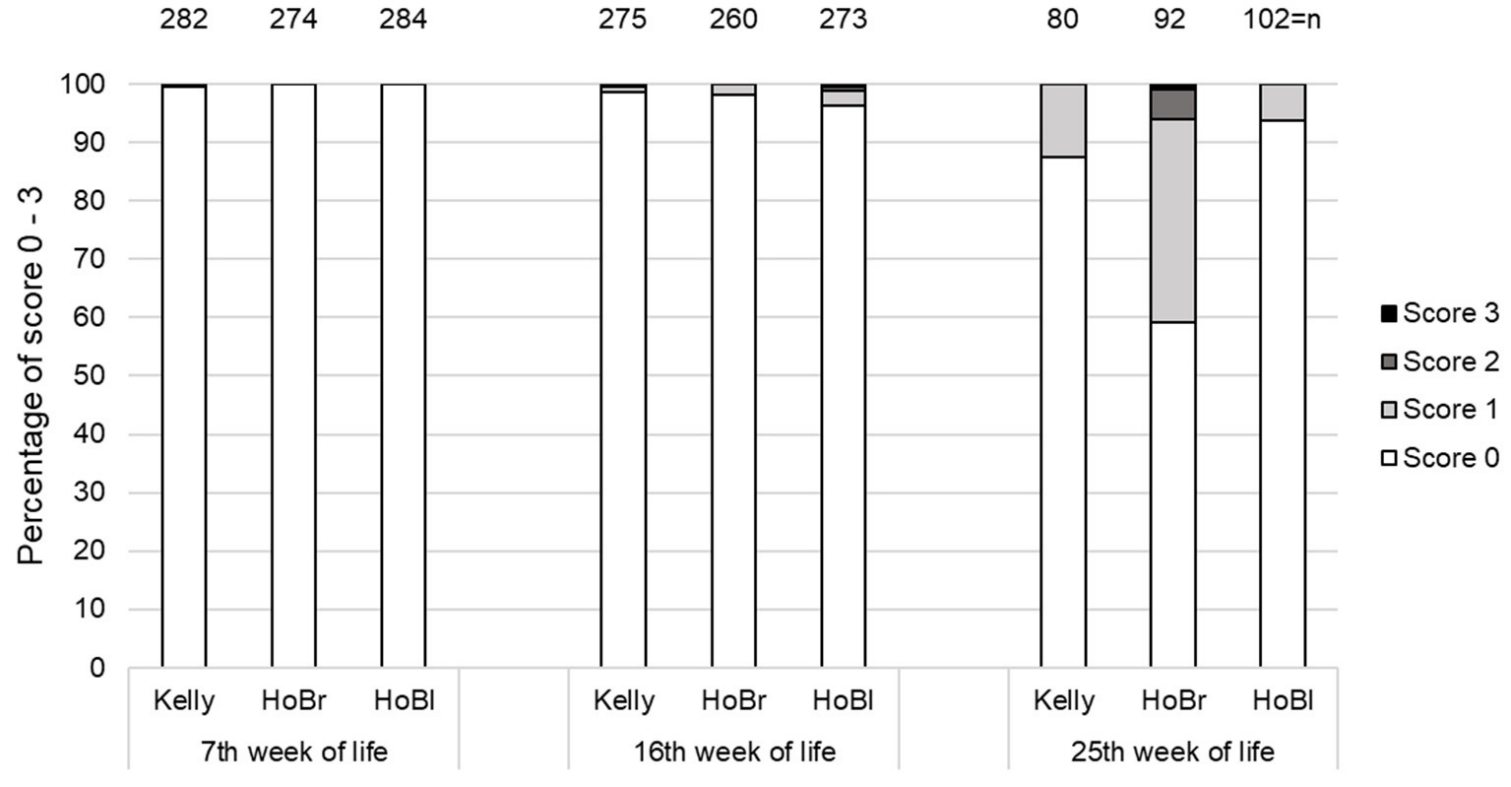
Percentages of turkeys with different gait scores (score 0 = normal, score 1 = slight abnormality, score 2 = defined lameness, score 3 = unable to walk) at three scoring times in three strains (Kelly = Kelly BBB, HoBr = Hockenhull Bronze, HoBl = Hockenhull Black) over three fattening batches (cycles), each with two groups per strain, n = total number of individuals assessed.

Except for Hockenhull Bronze in the 25th week of life, walking ability was mostly not impaired, but from the 16th week of life onwards score 3 was found once in each strain and score 2 in 0.8% (HoBl) and 5% of birds (HoBr). Thus, turkeys with impaired walking ability predominantly showed only slight abnormalities (score 1; Figure 4). Kelly was significantly less affected than HoBr, but not than HoBl (Table 4). No significant interaction between “strain” and “week of life” was found, whereas a significant effect of the latter was detected (*p* < 0.01).

Significantly more abnormal leg positions were detected in Kelly turkeys (*n* = 638) than in HoBl (*n* = 659), but not than in HoBr (*n* = 626; Table 4). They comprised mainly x-shaped legs and no o-shaped legs (definitions see Table 2). Some malpositions were already visible in the 7th week, with a marked increase until the 25th week of life. Overall, a significant effect of the factor “week of life” was detected (*p* < 0.01) but no significant interaction between “strain” and “week of life.”

**Figure 5:**
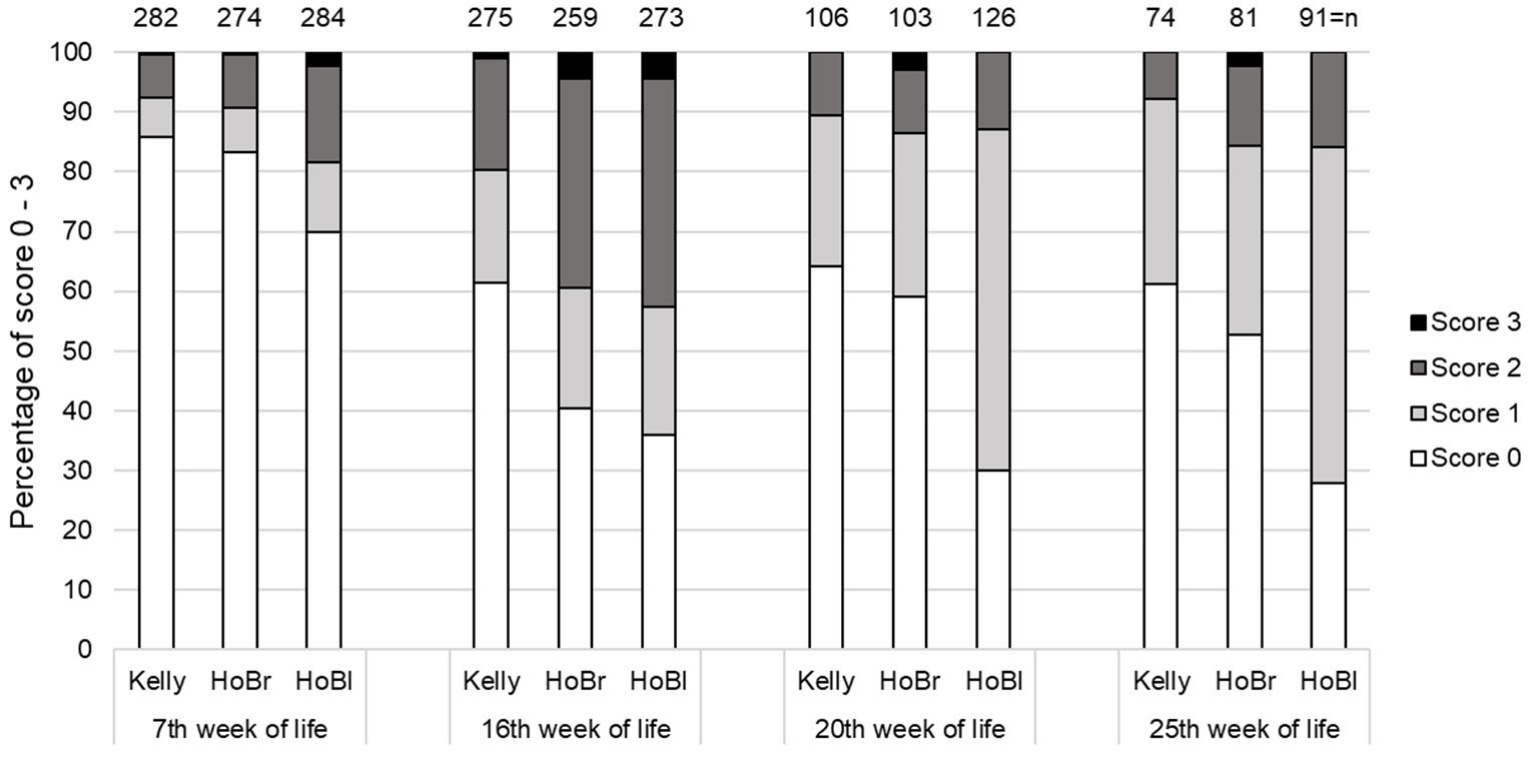
Percentages of turkeys with different footpad scores (score 0 = intact, score 1 = small necrotic spots, score 2 < 50% necrotic footpad, score 3 > 50% necrotic footpad) at four scoring times in three strains (Kelly = Kelly BBB, HoBr = Hockenhull Bronze, HoBl = Hockenhull Black) over three fattening batches (cycles), each with two groups per strain, n = total number of individuals assessed.

Footpad dermatitis affected Kelly significantly less than HoBl, but not than HoBr (Table 4), although HoBr showed larger increases of prevalences from the 7 to 16th week of life compared to Kelly (interaction: *p* = 0.02). Furthermore, a significant effect of “week of life” was detected (*p* < 0.01). Most alterations of the footpad were minor or medium (score 1 and score 2), from 7 to 57%, and only a small proportion (0–5%) were inflammations of larger areas of the footpad or toes (score 3; Figure 5).

Breast blisters occurred in 0–6% (*n* = 686) of slaughtered turkeys per strain and assessment time, with no significant differences between strains (Table 4). Breast buttons were more prevalent and occurred significantly more in Kelly (*n* = 244) than in HoBr (*n* = 247) and HoBl (*n* = 260; Table 4). For both alterations no significant interactions between “strain” and “week of life” or significant effects of the latter was found (*p* = 0.20–0.88). In total, only four birds (0.9–2%) (HoBr and Kelly) had breast buttons with a diameter of more than 2 cm (score 3). Up to 11% of Kelly turkeys showed smaller lesions (score 2), and the rest (0.9–5% of all lines) had very small lesions.

**Figure 6:**
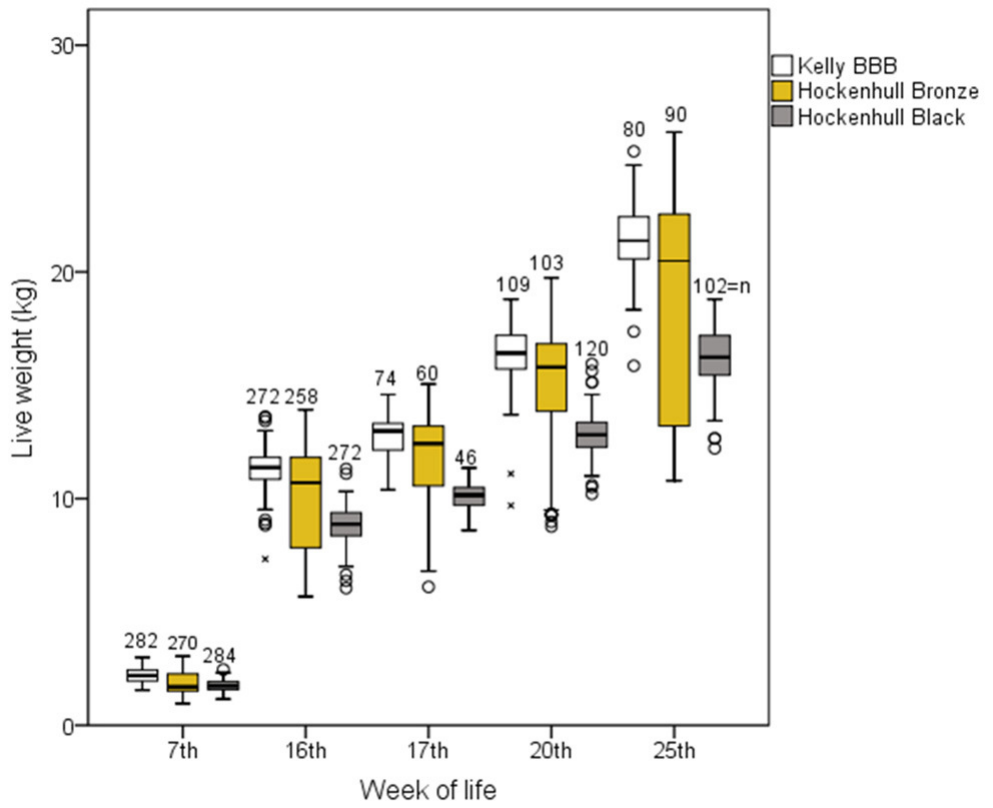
Live weight of three strains at five weighing times from three fattening batches (cycles), each with two groups per strain, with n (total number of weighed individuals) given; box plot with median, lower and upper quartile, minimum and maximum and outliers.

Concerning performance measures, Kelly achieved at all assessment times significantly higher average live weights (21.4 kg, 25th week) than HoBr (19.5 kg, 25th week) and HoBl (16.6 kg, 25th week; Figure 6; Table 5).

**Table 5:** Results of Kruskal-Wallis, Mann-Whitney-*U*-test and effect sizes (point-biserial correlation) regarding possible effects of “strain” on turkey performance in the 25th week (Kelly = Kelly Broad Breast Bronze, HoBr = Hockenhull Bronze, HoBl = Hockenhull Black).

	**Week**	**Kruskal-Wallis test**				**Mann-Whitney-*****U*****-test**		
		***n***	**χ^**2**^**	**df**	***p*-value**		***p*-value**	**r_**pb**_**
Live weight	25	80–102	92.43	2	<0.01	Kelly vs. HoBr	0.01	−0.35
						Kelly vs. HoBl	<0.01	−0.87
Carcass weight	25	72–90	78.64	2	<0.01	Kelly vs. HoBr	0.04	−0.30
						Kelly vs. HoBl	<0.01	−0.85
Utilization	25	49–67	1.41	2	0.49	–	–	–
						–	–	–

df, degrees of freedom; r_pb_, point-biserial correlation.

Furthermore, the average carcass weight was significantly higher in Kelly (16.6 kg, 25th week) than in HoBr (15.3 kg, 25th week) and HoBl (12.8 kg, 25th week) at most assessment times (Table 5). Utilization (Kelly and HoBl: 75%, HoBr: 76%) only differed at 20th week between Kelly and HoBl (Table 5). Feed conversion did not differ between strains (Kelly: 2.9:1, HoBr: 2.8:1, HoBl: 3.1:1 kg feed:kg live weight; *p* = 0.60, χ^2^ = 1.02, *n* = 5–6, reduced n due to lost documentation).

Average daily weight gain from day 1 until the 25th week of life was significantly higher in Kelly with 111 g than in HoBl with 84 g, but not statistically different from HoBr with 101 g (Table 6).

**Table 6:** Results of linear mixed models and effect sizes (point-biserial correlation) regarding possible effects of “strain” and “week of life”* on performance measures; significant interactions are stated in the text (HoBr = Hockenhull Bronze, HoBl = Hockenhull Black).

**Parameter**	**Line**	**Est**.	**Std. error**	**df**	***z*-value**	***p*-value**	**r_**pb**_**
Daily weight gain	Kelly vs. HoBr	−9.911	6.702	13.079	−1.479	0.163	−0.35
	Kelly vs. HoBl	−26.432	6.695	13.023	−3.948	0.002	−0.88
*n*	Kelly = 80, HoBr = 102, HoBl = 90						
Breast meat	Kelly vs. HoBr	−0.355	0.272	12.914	−1.306	0.214	−0.25
	Kelly vs. HoBl	−1.022	0.273	13.134	−3.743	0.002	−0.67
*n*	Kelly = 30, HoBr = 32, HoBl = 30						
Upper leg	Kelly vs. HoBr	−0.170	0.170	32.384	−0.997	0.326	−0.12
	Kelly vs. HoBl	−0.387	0.178	36.510	−2.175	0.036	−0.52
*n*	Kelly = 30, HoBr = 31, HoBl = 30						
Lower leg	Kelly vs. HoBr	−0.259	0.118	28.049	−2.201	0.036	−0.14
	Kelly vs. HoBl	−0.454	0.122	31.598	−3.705	0.001	−0.39
*n*	Kelly = 31, HoBr = 31, HoBl = 30						

*For analysis of daily weight gain a possible effect of “week of life” was not included.

Est., Estimate; Std. error, Standard error; df, degrees of freedom; r_pb_, point-biserial correlation.

Including all assessment times, the average breast weight after slaughter was significantly higher in Kelly (5.9 kg, 25th week) than in HoBl (4.7 kg, 25th week), but not compared to HoBr (5.7 kg, 25th week; Table 6). The same applied to the weight of the upper leg where Kelly reached 2.8 kg, HoBl 2.0 kg and HoBr 2.7 kg in the 25th week (Table 6). The weight of the lower leg was altogether higher in Kelly (2.1 kg, 25th week) than both in HoBl (1.4 kg, 25th week) and HoBr (1.9 kg, 25th week; Table 6). However, there were significant interactions between “week of life” and “strain”: HoBl showed a reduced increase in weight of upper and lower leg at the end of fattening period (*p* = 0.01–0.03). In case of breast weight, no significant interaction between “week of life” and “strain” were found, whereas a significant effect of “week of life” was found for breast weight, upper and lower leg weight (*p* < 0.01).

## Discussion

The expectation that strains differ in the extent of welfare problems depending on their growth potential (5, 36) was not confirmed in this study for the range of growth rates investigated. In general, almost all differences in animal welfare outcomes between the strains were of negligible (Φ < 0.10) to small effect size (Φ < 0.20–024). In contrast, other studies found strain differences, mostly comparing fast vs. slower growing strains, for cannibalism and feather pecking (10, 13, 14, 28) and partially for leg health (10, 44) vs. (28, 45) as well as breast skin health (10) vs. (21). While in the present study no statistical strain differences in terms of breast blisters were found, the slowest growing HoBl showed reduced prevalences of breast buttons with a small effect size (Φ = 0.20). HoBl additionally showed slightly fewer malpositions of the legs and reduced injury rates, but on the other hand had more cases of footpad dermatitis (again with small effect size: Φ = 0.24), for which a strain effect has not been reported before (14, 16, 19, 21, 45). Similarly, mixed results were found for HoBr turkeys that showed a rather similar growth rate compared to Kelly. They had slightly more problems concerning walking ability and plumage damage, but also less breast buttons than Kelly turkeys. Further, no statistical strain differences could be detected regarding the use of resources, contrary to the majority of earlier studies (10, 32, 44, 46) vs. (19, 28), and regarding mortality rate. Although it was numerically lower in HoBl, the variation between groups and batches was high and sample size on group level low. Thus, none of the studied strains showed clear benefits or disadvantages in terms of the birds' predisposition for welfare problems.

Concerning the general welfare and performance level of the monitored birds, the average mortality rates from 5.2 to 7.2% per strain during rearing and fattening can be regarded moderate compared to other study results (organic husbandry with different strains and not always including the rearing period) that ranged from 2 to 21% (10, 19, 47–49). In particular, considering the *Histomonas meleagridis* infection with commonly high mortalities of about 90–100% (50), it may even be deemed low. This may partly be explained by the immediate measures that were taken to limit the effects of the infection and that included closure of the free-range (3 weeks in batch one, 3 days in batch two and three), covering the litter with corrugated board and applying quicklime around the house.

Only three birds died as a result of cannibalism, which in all cases happened after single animals entered another group by jumping over the fence in the free-range area. Also, Buchwalder and Huber-Eichler (51) described that turkeys attack unknown animals more likely than animals from their own group. Despite lacking systematic behavioral observations of injurious pecking, it is very likely that apart from these very specific cases, no cannibalism occurred. Nevertheless, about one third of birds had injuries, mostly of superficial and small extent, which according to chance observations were caused by agonistic interactions between the males. This is an important difference to domestic fowl where injuries due to agonistic interactions can almost only be found around the combs. For turkeys, however, it is not clear whether injuries reported in the literature are related to cannibalism or agonistic behavior (10, 11, 13, 14, 21, 36). Only Spindler (15) explicitly states that in her study around 34% of the recorded injuries were due to cannibalism. Savory (52) described that often conspicuous bloody spots or damaged skin areas can trigger cannibalistic behavior. Therefore, it is noteworthy, that in our study, despite the presence of injuries from fighting, no outbreaks of cannibalism occurred. Comparisons of injury levels with other studies are further hampered by their partly lacking reports of the proportion of the different injury scores (13, 36, 49). However, the majority of researchers found roughly similar prevalences of injuries and also of plumage damage (10–15).

Similarly, although the majority of birds showed slight damage of single feathers, and a few birds had single lacking feathers, there was no indication of a manifest feather pecking problem in the monitored groups. Most of the damage was likely mechanically caused, because birds often came into contact with the equipment of the comparatively small pens or the pen partitions. In fact, this was the reason why in the last batch, the perches were removed in the last 3 weeks. In this context it is important to note that the study conditions were different from usual commercial husbandry conditions with commonly much larger groups than 50 birds and consequently larger absolute space allowances. On the other hand, it is possible that the small group sizes, especially during the rearing phase, contributed to the lack of cannibalism and feather pecking problems. This should be further investigated.

The use of perches during the day was, with around 4% of the observed time, generally low compared to other investigated slower growing turkeys [(32): 12% and 16%; (46): 10–31%]. The latter results only included young turkeys until the 12th week of life, and it is known that use of perches decreases with increasing live weight (46). Moreover, the observation time in comparable studies covered longer periods (32, 46). In this study, natural illumination levels in the winter garden were too low in the early morning and in the evening hours for reliable observations. A contribution to reduced perch use was probably the use of metal perches in the first batch; the acceptance by the animals was low. Additionally, the winter gardens, which were not available in the studies cited above, were intensively used (31%) and provided no perches. Furthermore, Berk et al. (12) found a lower use of winter gardens of about 9–11%. The free-range area, on the other hand, was less used (19%) then reported elsewhere [(10, 28, 32, 44): 36–94%], with the exception of Straßmeier (28) who found a reduced use in winter with 7–12%. According to Bergmann (10) and Straßmeier (28) the use of the free-range decreases with falling temperature. Since the access to the free-range in our study was mainly provided in fall and winter, this, together with the attractive winter gardens, might explain the lower use. No comparable figures regarding use of the feeding area are available. The time budget of 13–48% for foraging behavior, reported by Bircher and Schlup (53) for commercial lines, is higher than the 7% found here, but foraging behavior also occurs at other places than at the feeder in the pen where it was only recorded.

Leg health (walking ability, position of legs) was comparatively good in our study, considering reports of more than 50% of the turkeys having problems in this area (10, 27, 28). Our results may already reflect the recently increased breeding efforts to improve animal health and in particular leg health (54). Furthermore, Bergmann (10) found better leg health in winter. Thus, a seasonal effect might have contributed to the comparatively good results. Interestingly, the slightly worse walking ability of HoBr corresponded with a lower weight of the lower legs compared to Kelly despite rather similar live weights. On this line, Nestor et al. (55) found increased shank widths related to better walking ability.

Also footpad lesions were a less frequent problem (36–59% of birds) than in the majority of comparable studies that reported prevalences of more than 80% (14, 16–21). Still, there is room for improvement also regarding the results of the present study. The higher affliction of HoBl with the lowest live weights is in line with conclusions from Habig et al. (20) that genetic factors other than live weight influence footpad health. Alterations of the breast skin (3–10% of birds) were similarly or less prevalent compared to other studies with reported prevalences of 8–48% (10, 12, 21, 32–36).

Performance in general was high, considering the 100% organic diet fed from day 1 onwards. In other studies under conventional or organic conditions, Kelly reached mostly lower live and carcass weights (10, 34, 37, 47), whereas live weights of B.U.T Big 6 in comparable studies exceeded these results most of the time (10, 19, 34). However, in comparison breast weight of Kelly was lower (37). It is possible that the relatively low contents of methionine in the ration might have played a role.

## Conclusions

Despite partly differences in growth rate and predominantly slight differences in predispositions for welfare problems, no clear advantage or disadvantage of a specific strain could be identified. Overall, prevalences of animal welfare problems were mostly lower than in comparable studies and predominantly consisted of only minor alterations. Therefore, all monitored turkey strains appear to be suitable for rearing and fattening under organic conditions with 100% organic feed, given a good management, in terms of performance and animal welfare. However, it should be emphasized that group sizes were smaller than under usual commercial conditions. Therefore, it would be useful to conduct further investigations in larger groups to verify the results.

## Data Availability Statement

The raw data supporting the conclusions of this article will be made available by the authors, without undue reservation.

## Ethics Statement

The study was carried out in accordance with the German animal protection act (56). The protocol for the clinical monitoring and behavioral observations was reported to the competent authority (Nds. Landesamt für Verbraucherschutz und Lebensmittelsicherheit, LAVES) without objection and followed the “Guidelines for ethical treatment of animals in applied animal behavior and welfare research” (57).

## Author Contributions

AO designed the project together with UK (recording in live animals) and KR (recording in slaughtered animals). AO conducted data collection, analyses, and wrote the first draft of the manuscript. UK assisted in data analysis and interpretation of results. All authors contributed to the preparation of the manuscript and approved the submitted version.

## Conflict of Interest

The authors declare that the research was conducted in the absence of any commercial or financial relationships that could be construed as a potential conflict of interest.

## Supplementary Material

Supplementary File 1 Script for the calculations in *r*.
