# Supplementary material for: Suitability of Slower Growing Commercial Turkey Strains for Organic Husbandry in Terms of Animal Welfare and Performance
Source: Front Vet Sci. 2021 Jan 6;7:600846. doi: 10.3389/fvets.2020.600846 (PMC7873929; doi:10.3389/fvets.2020.600846)
Supplement: Supplementary File 1 — Script for the calculations in r. [file Data_Sheet_1.PDF]

```r
# Mixed model for binomial data with repeated measurements
library(lme4)
xModel <- glmer(X ~ Strain + Weekoflife + Strain:Weekoflife + (1|Batch/Group/Number),
data=turkey_final, family="binomial", control=glmerControl(optimizer="bobyqa"))
summary(xModel)
```

```r
# Mixed model for binomial data with single measurement
xModel <- glmer(X ~ Strain + Weekoflife + Strain:Weekoflife + (1|Batch/Group),
data=turkey_final, family="binomial",control=glmerControl(optimizer="bobyqa"))
summary(xModel)
```

```r
# Estimation of mean and confidence interval for binomial data
library(lme4)
xModel <- glmer(X ~ Strain + Weekoflife + Strain:Weekoflife + (1|Batch/Group/Number),
 data=turkey_final, family="binomial", control=glmerControl(optimizer="bobyqa"))
summary(xModel)
lsmeans(xModel, ~ Strain, type = "response")
```

```r
# Mixed model for metric data with repeated measurements
library(lme4)
library(lmerTest)
xModel <- lmer(X ~ Strain + Weekoflife + Strain:Weekoflife + (1|Batch/Group/Number),
data=turkey_final, control=lmerControl(optimizer="bobyqa"))
summary(xModel)
```

```r
# Mixed model for metric data with single measurement
library(lme4)
library(lmerTest)
xModel <- lmer(X ~ Strain + Weekoflife + Strain:Weekoflife + (1|Batch/Group),
data=turkey_final, control=lmerControl(optimizer="bobyqa"))
summary(xModel)
```

```r
# Test for normal distribution of residuals regarding metric data
library(car)
qqp(resid(xModel, type='pearson'))
library(moments)
skewness(resid(xModel))
kurtosis(resid(xModel))
```

```r
# Test for variance homogeneity regarding metric data
scatter.smooth(fitted (modelX), resid (modelX))
```

```r
# Approach for transforming data in the absence of normal distribution / variance homogeneity for single measurement
library(lme4)
library(lmerTest)
xModel <- lmer(log10(sqrt(X)) ~ Strain + Weekoflife + Strain:Weekoflife + (1|Batch/Group),
data=turkey_final, control=lmerControl(optimizer="bobyqa"))
summary(xModel)
```
